# Determination of Traces of Uranium and Thorium in Microelectronics Constituent Materials

**DOI:** 10.6028/jres.093.092

**Published:** 1988-06-01

**Authors:** Hideo Saisho, Masataka Tanaka, Koichi Nakamura, Eiji Mori

**Affiliations:** Toray Research Center, Inc., 2-1, Sonoyama 3-Chome, Otsu, Shiga 520, Japan; Sunric Co., Ltd., 2-8-9 Keihinzima, Ota-ku, Tokyo 143, Japan

## 1. Introduction

Because of the problem of soft errors due to *α*-particle emission by uranium and thorium [1], determination of trace amounts of these metals in microelectronics constituent materials has recently gained considerable attention. In the present paper, four different techniques: chemical analysis (CA), instrumental neutron activation analysis (INAA), glow discharge mass spectrometry (GD/MS), and inductively coupled plasma mass spectrometry (ICP/MS) were studied using high-purity aluminum samples A, B, and C.

## 2. Experimental

### 2.1 Chemical Analysis

Samples (1–5 g) were decomposed with nitric and hydrochloric acids, evaporated to dryness, dissolved in nitric acid and subjected to chemical analysis.

#### 2.1.1 Uranium Analysis [2,3]

With sodium nitrate and aluminum nitrate added into nitric acid solutions of samples, uranium was extracted twice with a carbon tetrachloride solution of tributyl phosphate (TBP). The organic phase was back- extracted thrice with dilute hydrochloric acid after being washed with a sodium nitrate solution and hydrochloric acid. The aqueous phase was evaporated to dryness. The residue was fused with the addition of a fusing mixture consisting of potassium carbonate, sodium carbonate and sodium fluoride. The fused product was set in a fluorophotometer to determine the uranium by measuring the fluorescence intensity at 556 nm.

#### 2.1.2 Thorium Analysis [4,5]

Aluminum nitrate and EDTA were added to nitric acid solutions of samples and the resulting solutions were extracted twice with mesityl oxide. The organic phase was back-extracted thrice with water. The aqueous phase, with acids added, was decomposed by heating. The residue was dissolved in hydrochloric acid and with Arsenazo III added to this solution, thorium was determined by measuring the absorbance at 660 nm.

### 2.2 INAA

About 5 g of each sample was irradiated for 24 h at a thermal neutron flux of 5.5×10^11^ neutrons cm^−2^ s^−1^. After cooling, the irradiated sample was counted for 10,000 s using a Ge (Li) detector. The cooling time was 6 to 8 days for uranium and 15 to 19 days for thorium. Uranium was determined by measuring *γ*-rays from 228 keV of ^239^Np and thorium from 312 keV of ^233^Pa.

### 2.3 GD/MS

The instrument used was a commercial GD/MS system (VG 9000, VG Isotopes). A pin-shaped sample was prepared for measurement. Discharge was conducted, using argon as the discharge gas, with a voltage of 1.0 kV and current of 2.0 mA. Quantitative analysis was performed at a mass resolution of 5400 with a data acquisition time of 75 to 150 s per isotope.

### 2.4 ICP/MS

Using a PlasmaQuad (VG Isotopes), measurements were taken with two kinds of sample solutions; one was for acid-decomposition, and the other for chemical separation of uranium and thorium from an acid-decomposed solution as in the case of chemical analysis.

In mass spectrometry, the isotopes of uranium and thorium measured were ^238^U and ^232^Th, respectively.

## 3. Results and Discussion

### 3.1 Accuracy and Precision

The accuracy and precision of the chemical analysis were confirmed using NBS reference materials. As can be seen from tables 1 and 2, our chemical analysis methods were found to have acceptable accuracy and precision.

### 3.2 Comparison of Results Obtained by Four Analytical Methods

Table 3 summarizes results obtained by chemical analysis, INAA, GD/MS and ICP/MS. So far as ICP/MS is concerned, results obtained by direct measurements of acid-decomposition solutions are omitted, because of unreliable nature of these results when the concentrations are lower than 100 ppb.

Three samples subjected to chemical analysis and INAA provide close agreement. Results of measurements by ICP/MS with Samples B and C are in close agreement with those by chemical analysis and INAA. However, with Sample A with the lowest concentrations of both uranium and thorium, somewhat higher analytical values have been obtained in ICP/MS than in INAA. The spectral signals observed in ICP/MS from Sample A’s uranium and thorium were apparently different from those of the blanks. On these grounds, the analytical values seem to have high accuracy.

The measured values by GD/MS are given in terms of ion beam ratios relative to that of an aluminum matrix. They are higher than the values obtained by the other three analytical methods with both Samples B and C. However, the ratio of the mean value of GD/MS to that of INAA falls in a range of 1.3–1.5 with both elements. Thus, by correction using this ratio or a relative sensitivity factor (RSF), satisfactory agreement can be obtained between the results from GD/MS analysis and the other three different analyses. If a more accurate RSF is obtained, it will become possible to make determinations of uranium and thorium very rapidly at concentration levels of 10 ppb or higher, as compared with the other three analytical methods.

## Figures and Tables

**Table 1:** Accuracy data for NBS reference materials

	This work (ppm)			Certified value (ppm)
	Run 1	Run 2	Mean	
1. Uranium				
SRM 616 (glass wafer)	0.068	0.069	0.068	0.0721 ±0.0013
2. Thorium				
SRM 614 (glass wafer)	0.75	0.74	0.74	0.748 ±0.006

**Table 2:** Precision data for the NBS 688 sample^a^

	Uranium (ppm)	Thorium (ppm)
Run 1	0.31	0.31
Run 2	0.33	0.31
Run 3	0.28	0.32
Run 4	0.30	0.30
Run 5	0.32	0.30
Mean	0.31	0.31
CV^b^	6%	3%

a SRM 688 (basalt rock): U. 0.37 ppm (non-certified). Th, 0.33±0.02 ppm (certified).

b Coefficient of variation [(standard deviation/mean)(100)]

**Table 3:** Analytical results for high-purity aluminum samples obtained by four different methods

		Sample A		Sample B		Sample C	
		U	Th	U	Th	U	Th
C A	(ppm)	0.001	<0.01	0.010	0.02	0.080	0.07
INAA	(ppb)	<1	1.6±0.8	10±2	14±2	82±4	79+4
GD/MS	(ppb)^a^	<5	<5	15±3	18±2	121 ± 17	119±8
ICP/MS^b^	(ppb)	2._2_	5._3_	12	12	79	84

a Ion beam ratio. Data not corrected using RSF. Mean ± standard deviation (5 determinations).

b Data obtained using the same solvent extraction procedure for CA.

## References

[1] May TC, Woods MH

[2] Science and Technology Agency (1982). Methods for Determination of Uranium.

[3] Sandell EB (1939). Colorimetric Determination of Traces of Metals.

[4] Grimaldi FS (1961). Treatise on Analytical Chemistry.

[5] Trofimova LA, Syromyatnikov NG (1965). Determination of Uranium, Thorium, and Zirconium with Arsenazo III without Chemical Separation. Zavodsk Lab (USSR).

